# Factors influencing QT interval prolongation during rifampicin-resistant tuberculosis treatment: a multicenter real-world study from China

**DOI:** 10.1186/s12879-025-11896-1

**Published:** 2025-12-12

**Authors:** Siyu Gao, Liping Zou, Peijun Tang, Qing Pan, Yan Liu, Wanli Kang, Jiaojie Ma, Qing Chen, Zhengyu Shi, Xianzhen Tang, Li Liang, Chunhui Guo, Juan Du, Qingdong Zhu, Song Yang, Zhanlin Chang, Zhouli Guo, Guihui Wu, Shenjie Tang

**Affiliations:** 1 Beijing Chest Hospital, Beijing Tuberculosis and Thoracic Tumor Research Institute, Capital Medical University, Beijing, 101149 China; 2 Department of Tuberculosis, Public Health Clinical Center of Chengdu, Jingjusi 18 Street, Jingjiang District, Chengdu, Sichuan 610061 China; 3 Department of Tuberculosis, the Fifth People’s Hospital of Suzhou, Jiangsu, China; 4 Department of Respiratory and Critical Care Medicine, Anqing Municipal Hospital, Anqing, Anhui China; 5 Taicang Affiliated Hospital of Soochow University, The First People’s Hospital of Taicang, Taicang, China; 6 Department of Tuberculosis, Haerbin Chest Hospital, Heilongjiang, China; 7 Department of Tuberculosis, Wuhan Pulmonary Hospital, Wuhan, Hubei China; 8 Department of Tuberculosis, The Fourth People’s Hospital of Nanning, Guangxi, China; 9 Department of Tuberculosis, Chongqing Public Health Medical Center, Chongqing, China; 10 Department of Tuberculosis, The Third People’s Hospital of Tibet Autonomous Region, Tibet, China

**Keywords:** MDR/RR-TB, QTc interval, Influencing factors, Adverse events, China

## Abstract

**Background:**

We sought to assess the factors influencing QTc interval prolongation in patients with multidrug- or rifampicin-resistant tuberculosis.

**Methods:**

We conducted a retrospective cohort study of admissions with multidrug-resistant or rifampicin-resistant tuberculosis as the primary diagnosis from nine regions in China between May 2018 and June 2020. Guided by directed acyclic graphs, we developed two multivariable logistic regression models to account for identified confounders and examine their impact on outcomes across different aspects. Results were represented as odds ratios with 95% confidence intervals. Statistical analysis was performed using RStudio.

**Results:**

Among the 1,421 patients included in the study, 1,293 (91.0%) had normal QTc intervals, while 128 (9.0%) exhibited prolonged QTc intervals. In treatment regimens-based adjusted models, treatment regimens containing bedaquiline and/or linezolid, baseline QTc prolongation (≥ 450 ms), hypertension, and pre-treatment anemia were significantly associated with an increased risk of QTc interval prolongation. Comorbidity-based adjusted models showed that baseline QTc interval prolongation, hypertension, pre-treatment anemia, and adverse events during treatment including gastrointestinal reactions, liver and kidney injury, and electrolyte imbalances were strongly correlated with QTc interval prolongation.

**Conclusions:**

Our study demonstrates that baseline QTc prolongation, hypertension and choice of treatment regimens significantly increase the risk of QTc interval prolongation in patients with multidrug- and rifampicin-resistant tuberculosis. Additionally, adverse events during treatment further elevate this risk and require careful monitoring.

**Supplementary Information:**

The online version contains supplementary material available at 10.1186/s12879-025-11896-1.

## Background

Tuberculosis (TB) is once again the leading cause of death from a single infectious agent worldwide, and the emergence of multidrug- and rifampicin-resistant tuberculosis (MDR/RR-TB) has exacerbated the severe public health crisis facing the world today [1]. As reported in the Global Tuberculosis Report 2024, it is estimated that in 2023, China had approximately 29,000 new MDR/RR-TB cases, accounting for 6.8% of global cases [2]. However, the treatment success rate for MDR/RR-TB in China stands at just 66%, which is considerably below the global average [2]. This highlights the critical role of MDR-TB treatment in controlling and reducing the TB burden.

Thanks in part to the advent of new drugs, global treatment success rate for MDR/RR-TB has improved over the past decade. According to the World Health Organization (WHO), the global average treatment success rate for MDR/RR-TB increased from 48% to 68% [2–4]. Since the WHO published the classification of second-line drugs for MDR/RR-TB in 2019, treatment regimens named BPaLM containing bedaquiline (Bdq), pretomanid, linezolid (Lzd), and moxifloxacin have shown better end-of-treatment outcomes in Belarus, South Africa, and Uzbekistan [5]. According to reports from South Africa, the BPaL regimen, which includes Bdq, pretomanid, and Lzd, along with other WHO-recommended second-line anti-TB drugs, has also improved treatment outcomes for drug-resistant TB [6].

Despite significant advances in clinical efficacy, treatment regimens for MDR/RR-TB remain more complex, longer in duration, and are associated with a higher risk of adverse events (AEs) compared to drug-sensitive TB. Of particular concern is the higher risk of QTc interval prolongation in TB patients. Prolongation of QTc intervals can result in life-threatening arrhythmias, such as torsades de pointes (TdP) [7, 8], which is a major AEs during TB treatment. TdP is a temporary arrhythmia that usually resolves on its own, but its attacks can occur rapidly and violently, potentially in some cases leading to sudden death [9]. Overall, TdP is relatively rare in large studies containing Bdq or other treatments [10, 11], but individual case reports have shown that it can occur in combination with other QT-causing drugs or electrolyte abnormalities [12]. Current research suggests that prolonged QTc is a key predictor of the occurrence of TdP and ventricular fibrillation [7, 13]. Studies have shown that MDR/RR-TB drugs themselves can prolong the QTc interval [9]. In a large multinational cohort with extended Bdq/Dlm use (*n* = 2,775), QT prolongation occurred in 20.9% (QTc>450ms) [14]. A South African cohort of patients receiving Bdq reported QTc >500 ms in 4.3% (*n* = 18) and QTc change >60 ms in 26.2% (*n* = 110), with no arrhythmias or related deaths [11]. In addition, the risk is further increased if there are other factors that may also contribute to the prolongation of the QTc interval.

Current guidance recommends an electrocardiogram (ECG) at baseline and at approximately weeks 2, 12, and 24, with at least monthly monitoring thereafter as clinically indicated. Against this practice backdrop, evidence on which patient- and treatment-related factors drive QTc prolongation in rifampicin-resistant tuberculosis (RR-TB) remains limited [15]. Therefore, this research aims to explore potential influencing factors associated with QTc interval prolongation. By integrating patient characteristics, laboratory data, and key variables before and after treatment, we seek to enhance the understanding of relevant risks. Based on the available evidence and our findings, we provide insights for managing QTc interval prolongation in patients with MDR/RR-TB during treatment.

## Methods

### Study population

Data were collected between May 2018 and June 2020, at tertiary care hospitals in nine regions in China, covering a wide geographical area (Figure S1). According to the National Tuberculosis Program (NTP) guidelines, patients would be assessed for the presence of TB and drug resistance by sputum smear microscopy, sputum culture and phenotypic susceptibility testing (DST) and molecular or genotypic testing such as Xpert MTB/RIF. The study finally included laboratory-confirmed MDR/RR-TB patients by rapid molecular, genotypic techniques or microbiology. Study setting: In 2018, Bdq was introduced into routine programmatic use in China within an active drug-safety monitoring framework, with ECG-based QTc surveillance recommended for regimens containing QT-prolonging agents. Therefore, enrolled patients were routinely treated with Bdq- and/or Lzd-containing regimens in our study. Inclusion criteria: According to the World Health Organization’s definition of drug-resistant tuberculosis (DR-TB) [16], all TB patients with pulmonary lesions that were at least resistant to rifampicin were included in this study, encompassing cases of RR-TB, MDR-TB, extensively drug-resistant TB (XDR-TB) and pre-extensively drug-resistant TB (Pre-XDR-TB). Exclusion criteria: (1) age < 18 years old; (2) refusing treatment; (3) missing medical record information; (4) patients without pre-treatment ECG or any during-treatment ECG; (5) congenital long QT syndrome.

### Data collection and study design overview

**Fig. 1:**
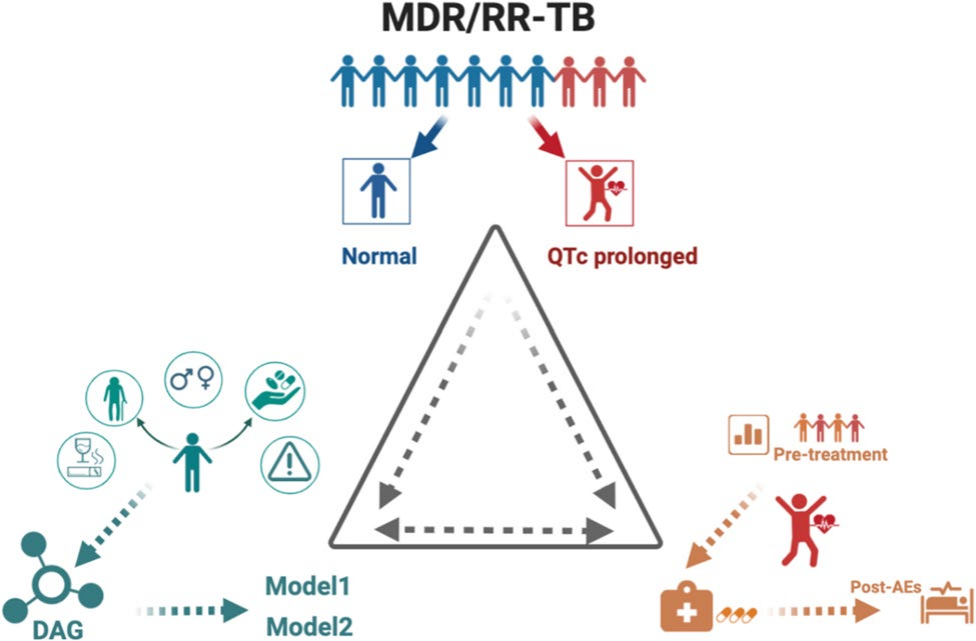
Study Design Overview

Medical practitioners who had undergone uniform training collected demographic information, clinical characteristics, treatment regimens, and laboratory test results by using the electronic medical system. This study employed a retrospective observational approach, examining populations with both prolonged and normal QTc intervals within a structured model. The research is grounded in evidence from previous studies on risk factors for QTc interval prolongation and further analyzes influencing factors in patients with MDR/RR-TB at three key stages: before treatment, during treatment, and after treatment (Fig. 1).

This article reviewed populations with prolonged QTc intervals and normal QTc intervals in longitudinal observational studies of MDR/RR-TB (top of triangle). The article focused first on evidence for risk factors for prolonged QTc intervals from relevant studies (lower left corner); the analyzes then considered the results of the studies according to process of studies (lower right corner).

### Ethical approval

The study complied with the principles outlined in the Declaration of Helsinki and received approval from the Ethics Committee of the Public Health Clinical Center of Chengdu, which served as the leading institution (Approval No. YJ-K2022-81-01). As a retrospective cohort study, the requirement for signed informed consent was waived in accordance with the Measures for the Ethical Review of Life Sciences and Medical Research Involving Humans, which permits the waiver of informed consent for minimal-risk research using anonymized data [17, 18].

### Definitions

Height and weight were directly measured by trained medical staff. Ethnicity was categorized into Han and others. Comorbidities:Critical cardiopathy included coronary heart disease, life-threatening arrhythmias, heart failure, cardiogenic shock, and severe valvular heart disease. Immunocompromised diseases included diseases of the autoimmune system, Acquired Immune Deficiency Syndrome (AIDS), organ transplantations and certain inherited immunodeficiencies, as well as other diseases treated with immunosuppressive therapy. Drug-related definitions: The treatment protocol for RR-TB patients was formulated according to WHO and Chinese guidelines as well as individualized factors [19, 20]. A retreatment patient was identified as someone who had undergone anti-TB drug therapy for over one month. And, “the treatment” refers to the MDR/RR-TB regimen initiated during the current study period. Cavity closure based on Computed Tomography (CT) examination. Laboratory definitions: Anemia (mild) is characterized by a hemoglobin (Hb) concentration of 10–11.9 g/dL in females and 11–12.9 g/dL in males, moderate as an Hb concentration of 7–9.9 g/dL, and severe as an Hb concentration below 7 g/dL. Leukopenia (mild) is defined as leukocyte count less than the lower limit of normal (LLN) down to 4.0 × 10^9^/L, moderate as 3.0–4.0 × 10^9^/L and severe as a count less than 3.0 × 10^9^/L. Thrombocytopenia is graded as a platelet count less than LLN down to 100 × 10^9^/L. Liver injury (mild) is defined as ALT < 3 times the upper limit of normal (ULN), moderate as ALT 3–5 times ULN, and severe as ALT ≥ 5 times ULN. Hypoproteinemia is defined as a total plasma protein level below 6.0 g/dL and a plasma albumin level below 3.0 g/dL. Kidney injury is defined as an elevation in serum creatinine levels. Hyperuricemia is defined as a serum uric acid level exceeding 420 µmol/L in males and 360 µmol/L in females.

### Study outcomes and QTc interval measurement

The main outcome of our study was the prolongation of the QTc interval observed during hospitalization or outpatient follow-up (every two weeks) in MDR/RR-TB patients received regular treatment during the study period. All patients’ QTc intervals were measured using a calibrated 12-lead ECG device, with measurements performed under standardized conditions to ensure consistency. The ECG readings were analyzed by trained medical personnel. QTc intervals were calculated using both Bazett and Fridericia correction formulas to adjust for heart rate variations, consistent with their recognized reliability in clinical literature. A QTc interval value > 450 ms was defined as abnormal for both sexes to ensure consistent classification across participating centers. QTc was further categorized as 450–499 ms and ≥ 500 ms. ECG results were collected before and after the start of treatment, and any prolongation of the QTc interval (non-environmentally induced transient prolongation of the QTc interval confirmed by a repeat ECG on the same day) will be considered to meet the study outcome. For cases with suspected QTc interval prolongation, a second trained medical professional independently reviewed the ECG results. Evaluations were performed monthly or semimonthly after the start of treatment.

### Assessment of potential confounders

By constructing a Directed Acyclic Graph (DAG) in a browser-based environment (DAGitty: https://www.dagitty.net, [21] we evaluated potential confounders and identified two adjustment models. Based on the characteristics of the study, prior knowledge, and expert consensus, the variables considered for inclusion in the DAG were treatment regimen [14, 22], comorbidities [23–26], age [27], gender [27], smoking [28], alcohol abuse [29], body mass index (BMI), post-treatment AEs, disease severity, initial treatment or retreatment, and pre-treatment QTc interval prolongation. We identified the minimal adjustment sets to estimate the total causal effect of treatment regimen and comorbidities on QTc interval prolongation.

### Statistical analysis

All analyses were performed using RStudio version 2024.4.1. Random forest regressions were performed to fill in missing values using R studio version, and variables with missing values > 10% were excluded. Descriptive analysis for continuous data was performed by reporting mean ± standard deviation or median, while categorical data were summarized as percentages. Univariate analysis of categorical variables was performed using Pearson’s chi-square test, and independent-sample t-tests were used for continuous variables. Factors with a *p*-value of less than 0.05 in the univariate analysis were incorporated into the multivariate model. After adjusting for potential confounders identified in the minimal adjusted sets, multivariate logistic regression was employed to examine the independent risk of QTc interval prolongation by treatment regimens and comorbidities. The direct and total effects were expressed as odds ratios (OR) with 95% confidence intervals (CIs). Results with a *p*-value < 0.05 were considered statistically significant.

## Results

### Demographics and clinical characteristics in patients with MDR/RR-TB

A total of 1,421 patients with MDR/RR-TB were studied: 1,293 (91.0%) had normal QTc intervals and 128 (9.0%) exhibited prolonged QTc intervals (Table 1). The two groups did not differ significantly in age distribution, BMI, disease duration, ethnicity, smoking status, or alcohol consumption. However, with a prespecified uniform QTc threshold of > 450 ms applied to both sexes, the prolonged-QTc group had 33.6% women vs. 28.8% in the normal-QTc group. The prolonged-QTc group also had more retreatment cases (64.1% vs. 56.8%), shorter stature (1.67 m vs. 1.70 m), lower weight (56.8 kg vs. 59.8 kg), more farmers (20.3% vs. 12.7%) and fewer unemployed (46.9% vs. 60.1%). Among complications, diabetes was the most frequent comorbidity in the study population. Prolonged QTc intervals group had a higher incidence of hypertension (14.8% vs. 4.3%) and viral hepatitis/carrier (6.3% vs. 3.9%). Additionally, during the observation period, normal group exhibited more clinical symptoms: fever 25.8% vs. 14.1% (*p* = 0.0048), night sweats 33.6% vs. 7.8% (*p* < 0.001), asthenia 39.9% vs. 15.6% (*p* < 0.001), weight loss 35.7% vs. 15.6% (*p* < 0.001), chest pain 30.9% vs. 14.1% (*p* < 0.001), and shortness of breath 38.2% vs. 18.8% (*p* < 0.001).

**Table 1:** Demographics and clinical characteristics in patients with MDR/RR-TB

	QTc<450(*n* = 1293)	QTc>450(*n* = 128)	*P* value
Gender			
Male	921 (71.2%)	85 (66.4%)	0.297
Female	372 (28.8%)	43 (33.6%)	
Age, years			
< 40	577 (44.6%)	63 (49.2%)	0.603
40–59	480 (37.1%)	43 (33.6%)	
≥ 60	236 (18.3%)	22 (17.2%)	
Height, m			
Mean (SD)	1.70 (0.0758)	1.67 (0.0751)	< 0.001
Median [Min, Max]	1.71 [1.10, 1.88]	1.68 [1.46, 1.81]	
Weight, kg			
Mean (SD)	59.8 (11.3)	56.8 (8.89)	< 0.001
Median [Min, Max]	60.0 [30.0, 169]	55.5 [40.0, 80.0]	
BMI, kg/m^2^			
Mean (SD)	20.8 (3.38)	20.3 (2.70)	0.0932
Median [Min, Max]	20.8 [10.8, 62.1]	20.1 [13.7, 27.2]	
Ethnic group			
Han	1213 (93.8%)	122 (95.3%)	0.628
Other ethnic groups	80 (6.2%)	6 (4.7%)	
Educational levels			
Illiterate	29 (2.2%)	5 (3.9%)	< 0.001
Primary school	112 (8.7%)	16 (12.5%)	
Middle school	378 (29.2%)	66 (51.6%)	
College or higher	302 (23.4%)	39 (30.5%)	
Unknown	472 (36.5%)	2 (1.6%)	
Occupation			
Unemployed	777 (60.1%)	60 (46.9%)	0.00708
Farmer	164 (12.7%)	26 (20.3%)	
Worker	50 (3.9%)	6 (4.7%)	
Civil servant	65 (5.0%)	2 (1.6%)	
Service industry	38 (2.9%)	3 (2.3%)	
Other	184 (14.2%)	28 (21.9%)	
Unknown	15 (1.2%)	3 (2.3%)	
Province			
Sichuan	319 (24.7%)	65 (50.8%)	< 0.001
Heilongjiang	438 (33.9%)	0 (0%)	
Anhui	29 (2.2%)	22 (17.2%)	
Beijing	164 (12.7%)	7 (5.5%)	
Guangxi	34 (2.6%)	1 (0.8%)	
Jiangsu	77 (6.0%)	2 (1.6%)	
Hubei	204 (15.8%)	30 (23.4%)	
Chongqing	27 (2.1%)	1 (0.8%)	
Xizang	1 (0.1%)	0 (0%)	
Residence			
Rural	487 (37.7%)	64 (50.0%)	0.00836
Urban	806 (62.3%)	64 (50.0%)	
TB duration (months)			
Mean (SD)	20.8 (3.38)	20.3 (2.70)	0.0932
Median [Min, Max]	20.8 [10.8, 62.1]	20.1 [13.7, 27.2]	
TB exposure history	103 (8.0%)	21 (16.4%)	0.00219
Alcohol consumption	261 (20.2%)	25 (19.5%)	0.952
Smoking	388 (30.0%)	40 (31.3%)	0.848
Initial treatment/Retreatment			
Initial Treatment	558 (43.2%)	46 (35.9%)	0.138
Retreatment	735 (56.8%)	82 (64.1%)	
Comorbidity			
Hypertension	56 (4.3%)	19 (14.8%)	< 0.001
Diabetes	254(19.6%)	13(10.2%)	0.0123
COPD	48 (3.7%)	4 (3.1%)	0.928
Critical cardiopathy	44 (3.4%)	3 (2.3%)	0.704
Tumor	14 (1.1%)	0 (0%)	0.475
Immunocompromised diseases	64 (4.9%)	7 (5.5%)	0.965
Viral hepatitis/carrier	51 (3.9%)	8 (6.3%)	0.31
Chronic kidney disease	11 (0.9%)	3 (2.3%)	0.245
Clinical characteristics			
Cough	1139 (88.1%)	107 (83.6%)	0.182
Expectoration	1043 (80.7%)	97 (75.8%)	0.227
Fever	333 (25.8%)	18 (14.1%)	0.0048
Hemoptysis	246 (19.0%)	28 (21.9%)	0.508
Night sweats	435 (33.6%)	10 (7.8%)	< 0.001
Asthenia	516 (39.9%)	20 (15.6%)	< 0.001
Weight decreased	462 (35.7%)	20 (15.6%)	< 0.001
Chest pain	399 (30.9%)	18 (14.1%)	< 0.001
Shortness of breath	494 (38.2%)	24 (18.8%)	< 0.001

*Abbreviation*: *BMI* Body Mass Index, *TB* Tuberculosis, *COPD* Chronic obstructive pulmonary disease

### Laboratory findings before and after treatment in MDR/RR-TB patients

The treatment regimens and the laboratory tests before and after treatment are summarized in Table 2. The regimens could be divided into a regimen containing Bdq, a regimen containing Lzd, and a regimen containing both Bdq and Lzd. Of 1,421 patients, 1,154 received a regimen containing Bdq, 906 received Lzd, and 244 received both drugs. Before treatment, anemia was present in 21.8% of the normal-QTc group and 37.5% of the prolonged-QTc group (*p* < 0.001); baseline hypoproteinemia was 32.0% vs. 33.6% (*p* = 0.791), and any liver injury 17.6% vs. 18.7% (*p* = 0.837). Notably, patients with prolonged QTc had more pre-treatment anemia (*p* < 0.001). On chest imaging, pulmonary lesions were nearly universal and typically bilateral (74.2% vs. 78.1% with bilateral involvement). During treatment, AEs increased, especially in the prolonged-QTc group, including anemia 50.0% vs. 24.8% (*p* < 0.001); leukopenia 24.2% vs. 13.4% (*p* = 0.0035); thrombocytopenia 20.3% vs. 8.0% (*p* < 0.001); any liver injury 63.3% vs. 35.5% (*p* < 0.001); kidney dysfunction 10.2% vs. 4.2% (*p* = 0.0047); hypothyroidism 26.6% vs. 9.8% (*p* < 0.001); electrolyte disorders 48.4% vs. 21.2% (*p* < 0.001); gastrointestinal reactions 33.6% vs. 12.7% (*p* < 0.001); peripheral neuritis 28.9% vs. 16.2% (*p* < 0.001); optic neuritis 18.0% vs. 7.9% (*p* < 0.001); and hyperuricemia 45.3% vs. 30.6% (*p* < 0.001).

**Table 2:** Laboratory findings before and after treatment in MDR/RR-TB patients

	QTc<450(*n* = 1293)	QTc>450(*n* = 128)	*P* value
Treatment Regimen			
including Bdq	1096 (84.8%)	58 (45.3%)	**< 0.001**
excluding Bdq	197 (15.2%)	70 (54.7%)	
Treatment Regimen			
including Lzd	789 (61.0%)	117 (91.4%)	**< 0.001**
excluding Lzd	504 (39.0%)	11 (8.6%)	
Treatment Regimen			
Bdq-Lzd combination	177 (13.7%)	67 (52.3%)	**< 0.001**
excluding Baq and Lzd	1116 (86.3%)	61 (47.7%)	
Before Treatment			
Anemia			
normal	1011 (78.2%)	80 (62.5%)	**< 0.001**
mild	240 (18.6%)	42 (32.8%)	
moderate	39 (3.0%)	5 (3.9%)	
severe	3 (0.2%)	1 (0.8%)	
Leukopenia			
normal	1225 (94.7%)	115 (89.8%)	0.0281
3000–4000	56 (4.3%)	9 (7.0%)	
<3000	12 (0.9%)	4 (3.1%)	
Thrombocytopenia			
normal	1266 (97.9%)	118 (92.2%)	**< 0.001**
<100,000	27 (2.1%)	10 (7.8%)	
QTc			
normal	904 (69.9%)	78 (60.9%)	0.0308
450-499ms	384 (29.7%)	48 (37.5%)	
≥ 500ms	5 (0.4%)	2 (1.6%)	
Liver Injury			
normal	1066 (82.4%)	104 (81.3%)	0.837
mild	202 (15.6%)	20 (15.6%)	
moderate	18 (1.4%)	3 (2.3%)	
severe	7 (0.5%)	1 (0.8%)	
Kidney dysfunction	21 (1.6%)	2 (1.6%)	1
Hypoproteinemia	414 (32.0%)	43 (33.6%)	0.791
Cavity location			
none	498 (38.5%)	48 (37.5%)	0.975
unilateral	508 (39.3%)	51 (39.8%)	
bilateral	287 (22.2%)	29 (22.7%)	
Lesion Location			
none	4 (0.3%)	0 (0%)	0.543
unilateral	329 (25.4%)	28 (21.9%)	
bilateral	960 (74.2%)	100 (78.1%)	
After Treatment			
Anemia			
normal	972 (75.2%)	64 (50.0%)	**< 0.001**
mild	221 (17.1%)	42 (32.8%)	
moderate	83 (6.4%)	20 (15.6%)	
severe	17 (1.3%)	2 (1.6%)	
Leukopenia			
normal	1120 (86.6%)	97 (75.8%)	0.00345
3000–4000	117 (9.0%)	22 (17.2%)	
<3000	56 (4.3%)	9 (7.0%)	
Thrombocytopenia			
normal	1190 (92.0%)	102 (79.7%)	**< 0.001**
<100,000	103 (8.0%)	26 (20.3%)	
Liver Injury			
normal	834 (64.5%)	47 (36.7%)	**< 0.001**
mild	398 (30.8%)	77 (60.2%)	
moderate	46 (3.6%)	3 (2.3%)	
severe	15 (1.2%)	1 (0.8%)	
Kidney dysfunction	54 (4.2%)	13 (10.2%)	0.00471
Hypothyroidism	127 (9.8%)	34 (26.6%)	**< 0.001**
Electrolyte disorders	274 (21.2%)	62 (48.4%)	**< 0.001**
Allergy	37 (2.9%)	6 (4.7%)	0.379
Gastrointestinal reactions	164 (12.7%)	43 (33.6%)	**< 0.001**
Psychological disorder	58 (4.5%)	8 (6.3%)	0.494
Peripheral neuritis	209 (16.2%)	37 (28.9%)	**< 0.001**
Optic neuritis	102 (7.9%)	23 (18.0%)	**< 0.001**
Hearing impairment	60 (4.6%)	8 (6.3%)	0.551
Hyperuricemia	396 (30.6%)	58 (45.3%)	**< 0.001**
Cavity closed			
No	1013 (78.3%)	83 (64.8%)	**< 0.001**
Yes	280 (21.7%)	45 (35.2%)	

*Abbreviation*: *Bdq* bedaquiline; linezolid, Lzd

### Univariate logistic regression of QTc interval prolongation in MDR/RR-TB patients

To screen risk factors for QTc prolongation, we conducted a univariate analysis of patients with and without QTc prolongation, as summarized in Table S1. We found that height, weight, residence, TB exposure history, hypertension, diabetes, fever, night sweats, asthenia, weight loss, chest pain, shortness of breath, pre-treatment anemia, pre-treatment leukopenia, pre-treatment thrombocytopenia, liver injury, anemia, leukopenia, thrombocytopenia, kidney injury, gastrointestinal reactions, peripheral neuritis, optic neuritis, hyperuricemia, electrolyte disorders, hypothyroidism, cavity closure, and the use of bedaquiline, linezolid and Bdq combined with Lzd in treatment regimens were associated with QTc intervals prolongation (Table S1).

### Assessment of factors influencing QTc interval prolongation by treatment regimen using an adjusted multivariable logistic regression

**Fig. 2:**
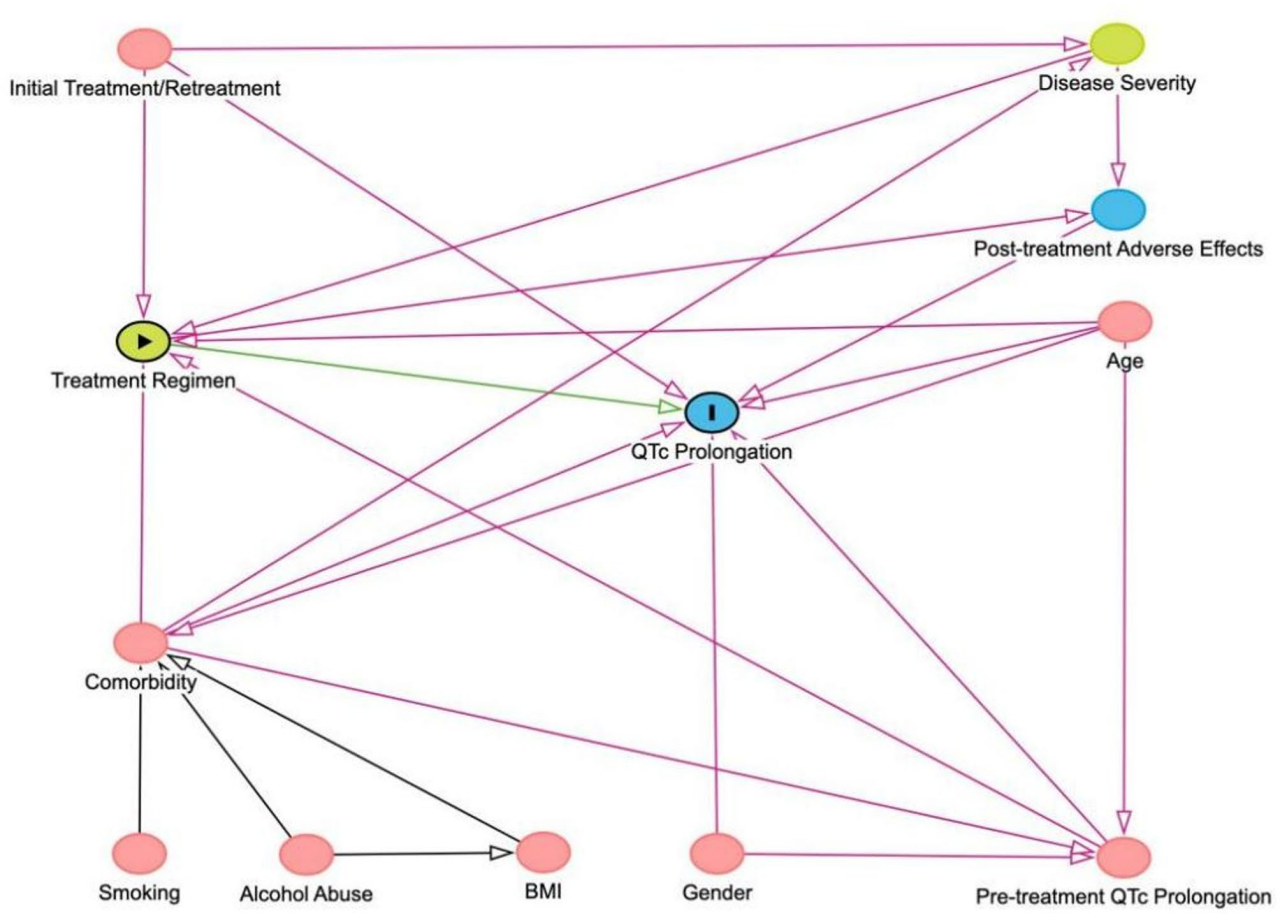
Directed acyclic graph (DAG) to distinguish the appropriate set of confounders for estimating the effect of treatment regimens on QTC interval prolongation

**Fig. 3:**
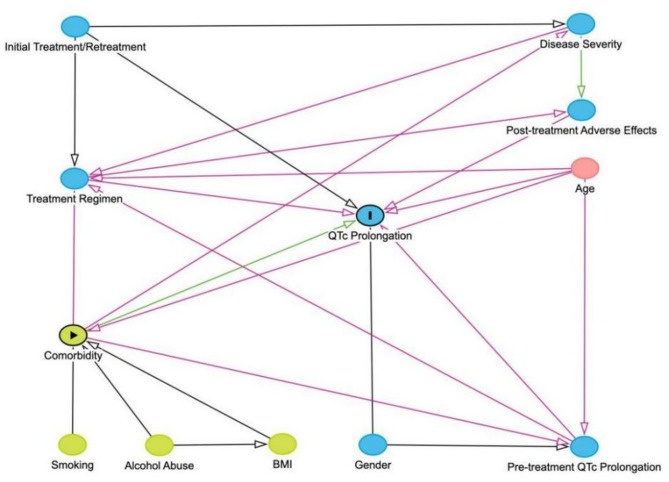
DAG to distinguish the appropriate set of confounders for estimating the effect of comorbidities on QTC interval prolongation

Considering the interactions between variables, two Directed Acyclic Graphs (DAGs) were constructed to illustrate the causal relationships between QTc interval prolongation and two main exposure factors: treatment regimens and comorbidities (Figs. 2 and 3). Guided by the DAGs, we conducted two multivariable logistic regression analyses to examine the effects of these factors on QTc interval prolongation. Original unadjusted multivariable logistic regression analysis was performed in Table S2.

The DAG showing different relationship pathways of treatment regimen and QTc interval prolongation.

The DAG showing different relationship pathways of comorbidities and QTc interval prolongation. DAG containing potential risk factors and confounders with their causal relations. Blue circles with black lining: outcome. Blue circles: ancestors of outcome. Pink circles: ancestors of exposure and outcome. Yellow circle: exposure. The green line with arrows indicates the only unidirectional causal pathway that comes from main exposure to main outcome.

In treatment regimens-based adjusted models, the treatment regimen containing Bdq (*OR* = 5.34, 95% *CI*: 3.48–8.20, *p* < 0.001), the treatment regimen containing Lzd (*OR* = 3.99, 95% *CI*: 2.06–7.75, *p* = 0.001), hypertension (*OR* = 6.64, 95% *CI*: 3.40–12.98.40.98, *p* < 0.001), and baseline QTc prolongation (≥ 500 ms) (*OR* = 9.33, 95% *CI*: 1.64–53.17, *p* = 0.012) were significantly associated with QTc interval prolongation during the treatment of MDR/RR-TB populations. Additionally, baseline QTc prolongation (450–499 ms) (*OR* = 1.73, 95% *CI*: 1.12–2.67, *p* = 0.013), pre-treatment anemia (*OR* = 2.00, 95% *CI*: 1.30–3.08, *p* = 0.002), and pre-treatment thrombocytopenia (*OR* = 2.48, 95% *CI*: 1.04–5.91, *p* = 0.040) were also identified as independent risk factors for QTc interval prolongation. Conversely, diabetes (*OR* = 0.38, 95% *CI*: 0.19–0.74, *p* = 0.004) was associated with lower odds of prolonged QTc interval (Table 3).

**Table 3:** Assessment of factors influencing for QTc interval prolongation by treatment regimen using an adjusted multiple logistic regression model*

Variable	B	SE	OR	Cl	Z	*P* value
Treatment regimen (Bdq)	1.675	0.219	5.34	3.48–8.2	7.666	< 0.001
Treatment regimen (Lzd)	1.385	0.338	3.99	2.06–7.75	4.095	0.001
Hypertension	1.893	0.342	6.64	3.4–12.98.4.98	5.529	< 0.001
Diabetes	−0.968	0.34	0.38	0.19–0.74	−2.844	0.004
COPD	−0.872	0.614	0.42	0.13–1.39	−1.42	0.156
Pre-treatment QTc prolongation (450–499)	0.55	0.221	1.73	1.12–2.67	2.494	0.013
Pre-treatment QTc prolongation (≥ 500)	2.233	0.888	9.33	1.64–53.17	2.515	0.012
Pre-treatment anemia	0.695	0.22	2	1.3–3.08	3.165	0.002
Pre-treatment thrombocytopenia	0.91	0.442	2.48	1.04–5.91	2.057	0.04

*DAG-based minimal adjustment set. The exposure variable was treatment regimen. We excluded the effects of adverse events after treatment, and included the following variables in our model: baseline QTc, age, comorbidities, initial treatment, and retreatment

### Assessment of factors influencing QTc interval prolongation by comorbidities using an adjusted multivariable logistic regression model

Using DAG-guided multivariable adjusted logistic regression models for further analyses to assess the influencing factors of comorbidities on QTc interval prolongation, we found that hypertension (*OR* = 4.19, 95% *CI*: 2.11–8.30, *p* < 0.001), pre-treatment QTc prolongation (450–499 ms) (*OR* = 3.46, 95% *CI*: 2.21–5.41, *p* < 0.001) and (≥ 500 ms) (*OR* = 8.36, 95% *CI*: 1.21–57.83, *p* = 0.031), and pre-treatment anemia (*OR* = 1.92, 95% *CI*: 1.24–3.00.24.00, *p* = 0.004) were associated with QTc interval prolongation. After the treatment, gastrointestinal reactions (*OR* = 1.79, 95% *CI*: 1.13–2.85, *p* = 0.014), liver injury (*OR* = 1.90, 95% *CI*: 1.21–3.00.21.00, *p* = 0.006), kidney injury (*OR* = 2.12, 95% *CI*: 1.02–4.39, *p* = 0.006) and electrolyte disorders (*OR* = 1.87, 95% *CI*: 1.16–2.99, *P* = 0.010) were also significant risk factors of QTc prolongation (Table 4).

**Table 4:** Assessment of factors influencing for QTc interval prolongation by comorbidities using an adjusted multiple logistic regression model*

Variable	B	SE	OR	Cl	Z	*P* value
Hypertension	1.433	0.349	4.19	2.11–8.3	4.1	< 0.001
Diabetes	−0.816	0.347	0.44	0.22–0.87	−2.354	0.019
COPD	−1.31	0.597	0.27	0.08–0.87	−2.194	0.028
Retreatment	0.342	0.217	1.41	0.92–2.15	1.577	0.115
Pre-treatment QTc prolongation (450–499)	1.241	0.228	3.46	2.21–5.41	5.439	< 0.001
Pre-treatment QTc prolongation (≥ 500)	2.123	0.987	8.36	1.21–57.83	2.152	0.031
Pre-treatment Anemia	0.654	0.226	1.92	1.24–3.24	2.901	0.004
Night sweats	−1.353	0.377	0.26	0.12–0.54	−3.585	< 0.001
Fever	−0.864	0.288	0.42	0.24–0.74	−3.003	0.003
Gastrointestinal reactions	0.583	0.237	1.79	1.13–2.85	2.461	0.014
Weight loss	−0.747	0.29	0.47	0.27–0.84	−2.576	0.01
Liver injury	0.643	0.232	1.9	1.21–3.21	2.771	0.006
Kidney injury	0.75	0.372	2.12	1.02–4.39	2.013	0.044
Electrolyte disorders	0.624	0.241	1.87	1.16–2.99	2.586	0.01
Optic neuritis	0.523	0.285	1.69	0.96–2.95	1.832	0.067

*DAG-based minimal adjustment set. The exposure variable was comorbidity. We excluded the effects of treatment regimen and severity of illness, and included the following variables in our model: baseline QTc, age, gender, comorbidities, initial treatment, and retreatment. Symptom variables (fever, night sweats and weight loss) were presented for adjustment only and not as protective effects

## Discussion

This multicenter retrospective cohort from nine hospitals in China assessed factors associated with QTc interval prolongation in patients with MDR/RR-TB. Guided by DAGs, we identified variables with relationships to treatment regimens and comorbidities and fit multivariable logistic regression models to estimate adjusted associations. The findings may provide a reference for influencing factors of QTc interval prolongation in the MDR/RR-TB population.

Drug-induced QT interval prolongation is a leading cause of treatment discontinuation [30]. Several anti-TB drugs, including Bdq, delamanid, clofazimine, and fluoroquinolones, are known to prolong QTc interval [9]. In our adjusted regression modeling, we assessed the association between treatment regimen and QTc interval prolongation and we found that regimens containing Bdq and Lzd were significantly associated with QTc prolongation. Bdq’s effect is well-documented via hERG-channel inhibition [31–35]. However, early studies on Lzd did not detect clinically meaningful QTc effects. In patients with bacterial infections receiving a single intravenous dose of 600 mg or 1200 mg Lzd, no significant QTc interval prolongation was observed [36]. Similarly, in studies of extensively DR-TB, although 87% of participants treated with Lzd experienced significant AEs, no cardiac-related AEs were reported [37].

In addition, Lzd combinations with drugs causing hypothyroidism (such as ethionamide, prothionamide, or para-aminosalicylic acid), can trigger hypokalemia and thus QTc prolongation [9, 38, 39]. Recently, the FDA has flagged a potential Lzd-QTc link in TB patients [40], and Li et al. found the BPaL regimen (including Lzd) had a greater QTc impact than single agents [41]. However, these results usually stem from the combined use of multiple drugs, rather than a direct Lzd effect. Thus, the observed association between linezolid-containing regimens and QTc prolongation should be interpreted with caution, does not constitute causal evidence, and does not imply that Lzd monotherapy is sufficient to prolong QTc.

In addition to TB treatment, our study also identified hypertension as a significant risk factor for QTc prolongation. The occurrence of QTc prolongation has been frequently described across various drugs, with certain antihypertensive drugs, such as loop diuretics, being known to induce electrolyte imbalances that contribute to QTc prolongation [42]. However, the impact of other common antihypertensive agents, such as the calcium channel blocker lacidipine and the angiotensin-converting enzyme inhibitor enalapril, on QTc intervals in animal models show conflicting results [42]. Previous studies have consistently linked hypertension to QTc prolongation [23, 43, 44], likely due to structural heart changes, especially left ventricular hypertrophy [43, 44]. Additionally, while the extent of left ventricular hypertrophy in hypertensive patients may resemble that seen in athletes, increased sympathetic nervous activity in these patients may disrupt ventricular repolarization, exacerbating QTc prolongation [45]. Therefore, hypertensive patients with TB and concurrent hypertension, QTc prolongation is likely driven by a combination of factors.

Diabetes is also a recognized QTc risk factor, with a reported 17.1% prevalence in Chinese diabetic patients [46]. Insulin resistance, the underlying pathophysiological mechanism of type 2 diabetes, has been associated with QTc prolongation [24, 47]. Moreover, hyperglycemia-induced cardiac stress can alter myocardial structure and prolong QTc [48]. Diabetic complications such as autonomic neuropathy and microvascular disease further raise risk [49–51]. However, research specifically focusing on the relationship between diabetes and QTc prolongation in TB patients remains limited. In our study, the link between diabetes and QTc prolongation was relatively weak, possibly due to the small number of diabetic patients (*n* = 13) in the prolonged-QTc group.

Also, anemia was identified as a potential contributor to QTc prolongation. It may indirectly affect QTc by impairing cardiac perfusion and metabolism [52–54]. Hypoxemia and myocardial ischemia are common causes of QTc prolongation in anemic patients [54, 55]. Anemia may also increase susceptibility to cardiovascular conditions that further prolong QTc [54]. While not a direct cause, anemia affects cardiac function and the QT interval through multiple pathways, warranting close monitoring during DR-TB treatment.

In MDR/RR-TB treatment, the influence of comorbidities cannot be overlooked. We refined the model to further explore the influence of comorbidities on QTc prolongation. We found that even after adjusting for regimens, conditions such as hypertension, anemia, gastrointestinal reactions, liver damage, kidney damage, and electrolyte imbalances remained strong risk factors. In addition to hypertension and diabetes, patients with cancer or other chronic diseases, who often receive QTc-prolonging drugs and are prone to gastrointestinal AEs, face heightened QTc risk due to induced electrolyte disturbances [1, 25, 26]. Similarly, liver and kidney impairment, as seen in hepatitis carriers and CKD patients, may prolong QTc by slowing drug metabolism and altering electrolytes [26, 56]. Cardiovascular disease is also a known risk factor [57]. Although these comorbidities theoretically raise QTc risk in TB patients, our multifactorial model showed no significant associations, likely due to low comorbidity prevalence or overly broad category definitions, limiting statistical power.

In addition, retreatment was also a possible risk factor for QTc prolongation in our study. Consistent with our findings, recent studies report that QTc >450 ms is more frequent in retreatment DR-TB patients and linked to poorer outcomes [58]. This may relate to long-term drug exposure, tolerance and altered immune function, highlighting the necessity for more stringent management of QTc prolongation in retreatment patients.

Evidence on the determinants of QTc prolongation remains heterogeneous. For instance, a Ugandan DR-TB cohort treated with Bdq-based regimens found higher BMI linked to increased QTc risk [59]. Conversely, another study showed higher BMI reduces Bdq exposure (Cmin, AUC), theoretically lowering QTc risk [60]. Yet, weight-based dose increases may lead to cumulative drug effects, potentially raising QTc risk [61]. Therefore, while there may be an association between BMI and QTc interval, this relationship does not appear to be directly causal. Similarly, findings also from Uganda, revealed that HIV infection was associated with a reduced likelihood of QTc prolongation [59]. Unfortunately, the small number of HIV-infected patients in our study precluded statistical analysis, and the correlation between HIV infection and QTc prolongation remains controversial. Some studies suggested that HIV suppresses QTc prolongation, while others found HIV patients on antiretroviral therapy increased the risk [59, 62, 63].

This study reveals the factors influencing QTc interval prolongation in MDR/RR-TB patients before and during treatment. Based on these findings, we recommend:

1. Treatment regimen selection: For patients with baseline risk factors associated with QTc prolongation, clinicians should prioritize regimens with a lower overall QT risk and avoid combining multiple QT-prolonging agents whenever alternatives of comparable efficacy exist.
2. Management of comorbidities during treatment: Clinicians should not only monitor the impact of regimens on QTc intervals but also give special attention to comorbidities and AEs including gastrointestinal reactions, liver damage, kidney damage, and electrolyte imbalances that may exacerbate QTc prolongation in the course of treatment.

Although this study provides important insights, several limitations must be acknowledged. Firstly, patients from less-developed regions were underrepresented, which may limit generalize broadly. As a retrospective cohort study, the data relied heavily on existing medical records, which may have led to omission or incomplete capture of key clinical information. While we adjusted for gender in multivariable analyses, the use of a uniform > 450 ms threshold for both sexes could overclassify prolonged QTc among women. Despite the long-term follow-up, there were inevitable instances of patient loss to follow-up, leaving only 128 QTc prolongation cases in the final analysis, which may weaken the reliability of causal inferences, particularly when risk assessment for certain comorbidities (e.g., diabetes mellitus) was performed. These signals should be considered exploratory, and therefore the results may not be widely generalizable. We also could not distinguish the impact of different dosages or drug combinations on QTc prolongation. Future research should involve larger, more diverse cohorts and explore dosage- and regimen-specific effects.

## Conclusion

This study identifies key factors influencing QTc interval prolongation in patients with MDR/RR-TB. Early identification of high-risk factors, combined with regular monitoring, could significantly reduce the risk of QTc prolongation in these patients. Future research should further explore the role of other potential contributors to QTc prolongation, especially for the interaction effect of different drug combinations, to develop safer individualized treatment strategies for MDR/RR-TB patients.

## Supplementary Information

Supplementary Material 1.

Supplementary Material 2.

Supplementary Material 3.

Supplementary Material 4.

## Data Availability

The datasets used and/or analysed during the current study are available from the corresponding author on reasonable request.

## Abbreviations

TB: Tuberculosis
RR-TB: Rifampicin-Resistant Tuberculosis
MDR-TB: Multidrug-Resistant Tuberculosis
QTc: Corrected QT Interval
TdP: Torsades de Pointes
Bdq: Bedaquiline
Lzd: Linezolid
BPaL: Bedaquiline, Pretomanid, and Linezolid
DAG: Directed Acyclic Graph
NTP: National Tuberculosis Program
WHO: World Health Organization
ECG: Electrocardiogram
XDR-TB: Extensively Drug-Resistant Tuberculosis
Pre-XDR-TB: Pre-Extensively Drug-Resistant Tuberculosis
ALT: Alanine Aminotransferase
ULN: Upper Limit of Normal
Hb: Hemoglobin
AIDS: Acquired Immune Deficiency Syndrome
CI: Confidence Interval
OR: Odds Ratio
