# Supplementary figures and images for: Factors influencing QT interval prolongation during rifampicin-resistant tuberculosis treatment: a multicenter real-world study from China
Source: BMC Infect Dis. 2025 Dec 12;26:31. doi: 10.1186/s12879-025-11896-1 (PMC12794395; doi:10.1186/s12879-025-11896-1)

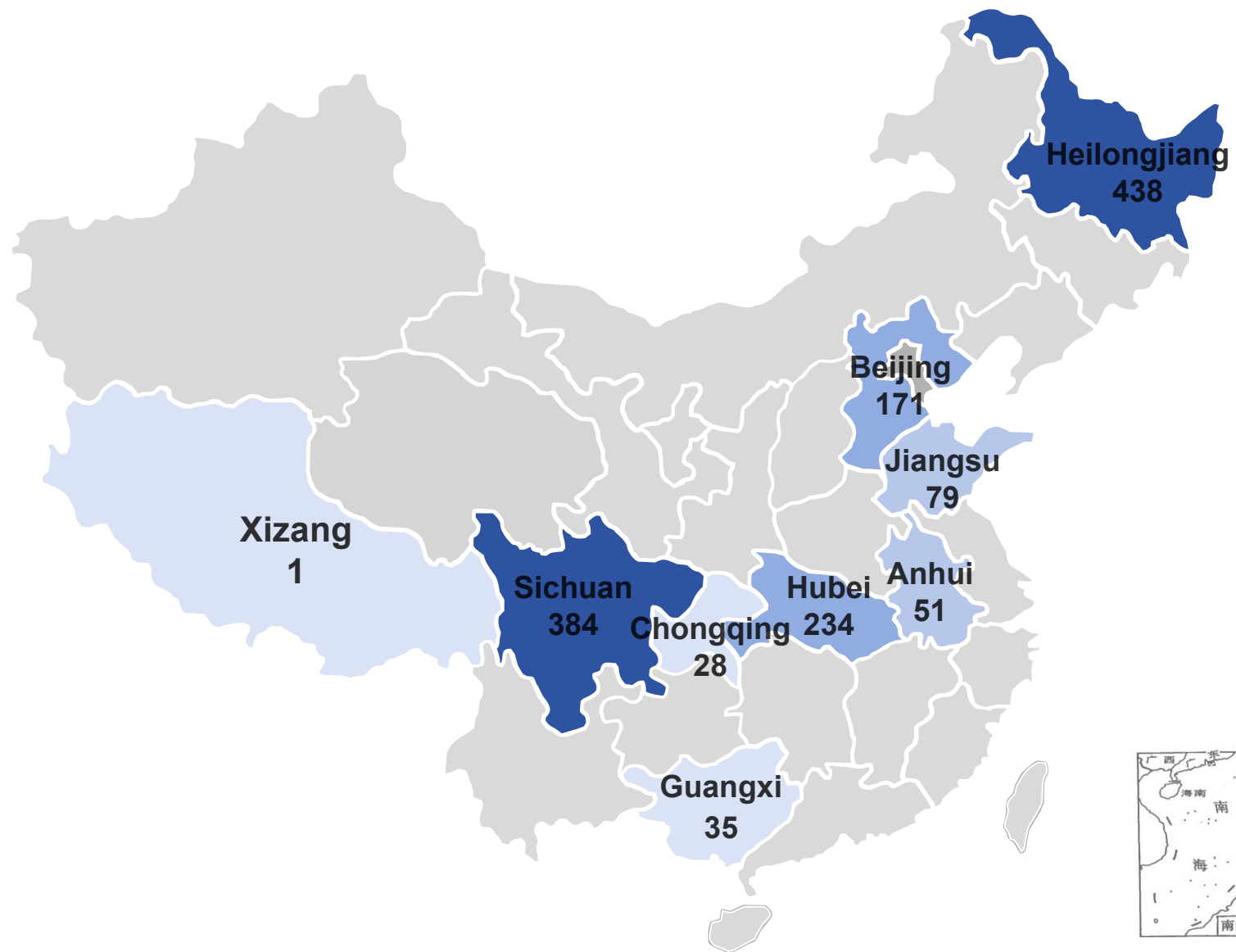

Supplement: Supplementary file 1 — Supplementary Material 1. [file 12879_2025_11896_MOESM1_ESM.pdf]
