# Supplementary material for: Factors influencing QT interval prolongation during rifampicin-resistant tuberculosis treatment: a multicenter real-world study from China
Source: BMC Infect Dis. 2025 Dec 12;26:31. doi: 10.1186/s12879-025-11896-1 (PMC12794395; doi:10.1186/s12879-025-11896-1)
Supplement: Supplementary file 2 — Supplementary Material 2. [file 12879_2025_11896_MOESM2_ESM.docx]

**Table S1. Univariate logistic regression of QTc interval prolongation in MDR/RR-TB patients**

|  | ***B*** | ***SE*** | ***OR*** | ***Cl*** | ***Z*** | ***P* value** |
| --- | --- | --- | --- | --- | --- | --- |
| Gender | 0.225 | 0.197 | 1.25 | 0.85-1.84 | 1.143 | 0.253 |
| age(40-59) | -0.198 | 0.207 | 0.82 | 0.55-1.23 | -0.955 | 0.34 |
| age(≥60) | -0.158 | 0.259 | 0.85 | 0.51-1.42 | -0.609 | 0.542 |
| Height | -3.971 | 1.144 | 0.02 | 0-0.18 | -3.473 | **0.001** |
| Weight | -0.027 | 0.009 | 0.97 | 0.96-0.99 | -2.992 | **0.003** |
| BMI | -0.042 | 0.03 | 0.96 | 0.9-1.02 | -1.42 | 0.156 |
| Ethnic group | -0.293 | 0.434 | 0.75 | 0.32-1.75 | -0.676 | 0.499 |
| Residence | -0.504 | 0.186 | 0.6 | 0.42-0.87 | -2.711 | **0.007** |
| TB exposure history | 0.819 | 0.26 | 2.27 | 1.36-3.77 | 3.151 | **0.002** |
| Hypertension | 1.348 | 0.284 | 3.85 | 2.21-6.72 | 4.753 | **<0.001** |
| COPD | -0.178 | 0.529 | 0.84 | 0.3-2.36 | -0.337 | 0.736 |
| Diabetes | -0.771 | 0.301 | 0.46 | 0.26-0.83 | -2.564 | **0.01** |
| Critical cardiopathy | -0.384 | 0.604 | 0.68 | 0.21-2.23 | -0.635 | 0.525 |
| Tumor | -13.264 | 388.97 | 0 | 0-Inf | -0.034 | 0.973 |
| Immunocompromised diseases | 0.105 | 0.409 | 1.11 | 0.5-2.48 | 0.257 | 0.797 |
| Viral hepatitis | 0.485 | 0.392 | 1.62 | 0.75-3.5 | 1.236 | 0.216 |
| Chronic kidney disease | 1.029 | 0.658 | 2.8 | 0.77-10.16 | 1.563 | 0.118 |
| Alcohol consumption | -0.041 | 0.233 | 0.96 | 0.61-1.52 | -0.176 | 0.86 |
| Smoking | 0.058 | 0.2 | 1.06 | 0.72-1.57 | 0.292 | 0.77 |
| Initial treatment/Retreatment | 0.303 | 0.193 | 1.35 | 0.93-1.98 | 1.571 | 0.116 |
| TB duration | -0.003 | 0.004 | 1 | 0.99-1 | -0.853 | 0.394 |
| Sputum AFB | 0.146 | 0.189 | 1.16 | 0.8-1.68 | 0.772 | 0.44 |
| Sputum culture | 0.103 | 0.329 | 1.11 | 0.58-2.11 | 0.312 | 0.755 |
| Cough | -0.373 | 0.254 | 0.69 | 0.42-1.13 | -1.469 | 0.142 |
| Expectoration | -0.288 | 0.218 | 0.75 | 0.49-1.15 | -1.32 | 0.187 |
| Fever | -0.751 | 0.262 | 0.47 | 0.28-0.79 | -2.867 | **0.004** |
| Hemoptysis | 0.175 | 0.225 | 1.19 | 0.77-1.85 | 0.779 | 0.436 |
| Night sweats | -1.789 | 0.335 | 0.17 | 0.09-0.32 | -5.347 | **<0.001** |
| Asthenia | -1.277 | 0.25 | 0.28 | 0.17-0.46 | -5.109 | **<0.001** |
| Weight loss | -1.099 | 0.25 | 0.33 | 0.2-0.54 | -4.394 | **<0.001** |
| Chest pain | -1.003 | 0.261 | 0.37 | 0.22-0.61 | -3.841 | **<0.001** |
| Shortness of breath | -0.986 | 0.234 | 0.37 | 0.24-0.59 | -4.22 | **<0.001** |
| Lesion Location(Unilateral) | 12.102 | 441.372 | 180270.64 | 0-Inf | 0.027 | 0.978 |
| Lesion Location(Bilateral) | 12.304 | 441.372 | 220643.75 | 0-Inf | 0.028 | 0.978 |
| Cavity location(Unilateral) | 0.041 | 0.211 | 1.04 | 0.69-1.58 | 0.193 | 0.847 |
| Cavity location(Bilateral) | 0.047 | 0.247 | 1.05 | 0.65-1.7 | 0.191 | 0.848 |
| Pre-treatment Anemia | 0.766 | 0.195 | 2.15 | 1.47-3.15 | 3.936 | **<0.001** |
| pre_Leukopenia | 0.711 | 0.318 | 2.04 | 1.09-3.8 | 2.236 | 0.025 |
| pre_Thrombocytopenia | 1.38 | 0.382 | 3.97 | 1.88-8.4 | 3.607 | **<0.001** |
| Pre-treatment QTc prolongation(450-499) | 0.371 | 0.193 | 1.45 | 0.99-2.11 | 1.918 | 0.055 |
| Pre-treatment QTc prolongation(≥500) | 1.534 | 0.845 | 4.64 | 0.88-24.29 | 1.815 | 0.069 |
| Pre-treatment Liver injury | 0.08 | 0.238 | 1.08 | 0.68-1.73 | 0.338 | 0.736 |
| Pre-treatment KidneyInjury | -0.039 | 0.746 | 0.96 | 0.22-4.15 | -0.053 | 0.958 |
| Pre-treatment hypoproteinemia | 0.071 | 0.196 | 1.07 | 0.73-1.58 | 0.364 | 0.716 |
| Liver injury | 1.141 | 0.192 | 3.13 | 2.15-4.56 | 5.934 | **<0.001** |
| Anemia | 1.108 | 0.188 | 3.03 | 2.09-4.38 | 5.889 | **<0.001** |
| Leukopenia | 0.727 | 0.222 | 2.07 | 1.34-3.2 | 3.277 | **0.001** |
| Thrombocytopenia | 1.08 | 0.243 | 2.94 | 1.83-4.74 | 4.454 | **<0.001** |
| Allergy | 0.513 | 0.45 | 1.67 | 0.69-4.03 | 1.138 | 0.255 |
| Kidney injury | 0.953 | 0.324 | 2.59 | 1.37-4.89 | 2.942 | **0.003** |
| Psychiatric disorder | 0.35 | 0.389 | 1.42 | 0.66-3.04 | 0.9 | 0.368 |
| Gastrointestinal reactions | 1.248 | 0.205 | 3.48 | 2.33-5.2 | 6.088 | **<0.001** |
| Peripheral neuritis | 0.746 | 0.209 | 2.11 | 1.4-3.18 | 3.568 | **<0.001** |
| Optic neuritis | 0.939 | 0.252 | 2.56 | 1.56-4.19 | 3.722 | **<0.001** |
| Hearing impairment | 0.315 | 0.388 | 1.37 | 0.64-2.93 | 0.811 | 0.418 |
| Hyperuricemia | 0.63 | 0.188 | 1.88 | 1.3-2.71 | 3.357 | **0.001** |
| Electrolyte disorder | 1.251 | 0.19 | 3.49 | 2.41-5.07 | 6.601 | **<0.001** |
| Hypothyroidism | 1.2 | 0.221 | 3.32 | 2.15-5.12 | 5.434 | **<0.001** |
| Cavity closed | 0.674 | 0.197 | 1.96 | 1.33-2.89 | 3.419 | **0.001** |
| Immunotherapy | -16.467 | 413.355 | 0 | 0-Inf | -0.04 | 0.968 |
| Nutritional therapy | -1.262 | 1.018 | 0.28 | 0.04-2.08 | -1.239 | 0.215 |
| Treatment regimen(Bdq) | -1.904 | 0.194 | 0.15 | 0.1-0.22 | -9.832 | **<0.001** |
| Treatment regimen(Lzd) | 1.916 | 0.32 | 6.79 | 3.63-12.72 | 5.979 | **<0.001** |
| Treatment regimen(Bdq+Lzd) | 1.935 | 0.195 | 6.93 | 4.73-10.15 | 9.945 | **<0.001** |

Abbreviation: OR, odds ratio; CI, confidence interval; ref, reference; BMI, body mass index;
