# Supplementary material for: Factors influencing QT interval prolongation during rifampicin-resistant tuberculosis treatment: a multicenter real-world study from China
Source: BMC Infect Dis. 2025 Dec 12;26:31. doi: 10.1186/s12879-025-11896-1 (PMC12794395; doi:10.1186/s12879-025-11896-1)
Supplement: Supplementary file 3 — Supplementary Material 3. [file 12879_2025_11896_MOESM3_ESM.docx]

**Table S2. Multiple logistic regression of QTc interval prolongation in MDR/RR-TB patients（unadjusted）**

|  | **B** | **SE** | **OR** | **Cl** | ***Z*** | ***P* value** |
| --- | --- | --- | --- | --- | --- | --- |
| Treatment regimen(Bdq) | 1.687 | 0.23 | 5.4 | 3.44-8.48 | 7.342 | <0.001 |
| Treatment regimen(Lzd) | 1.075 | 0.344 | 2.93 | 1.49-5.75 | 3.122 | 0.002 |
| Hypertension | 1.693 | 0.356 | 5.44 | 2.71-10.92 | 4.761 | <0.001 |
| Gastrointestinal reactions | 0.645 | 0.244 | 1.91 | 1.18-3.08 | 2.65 | 0.008 |
| Night sweats | -0.895 | 0.358 | 0.41 | 0.2-0.82 | -2.497 | 0.013 |
| Fever | -1.012 | 0.289 | 0.36 | 0.21-0.64 | -3.501 | <0.001 |
| Liver injury | 0.425 | 0.236 | 1.53 | 0.96-2.43 | 1.799 | 0.072 |
| Pre-treatment Anemia | 0.548 | 0.231 | 1.73 | 1.1-2.72 | 2.373 | 0.018 |
| Optic neuritis | 0.571 | 0.295 | 1.77 | 0.99-3.16 | 1.935 | 0.053 |
| Diabetes | -0.673 | 0.35 | 0.51 | 0.26-1.01 | -1.924 | 0.054 |
| Kidney injury | 0.791 | 0.382 | 2.21 | 1.04-4.66 | 2.071 | 0.038 |
| Electrolyte disorder | 0.465 | 0.25 | 1.59 | 0.98-2.6 | 1.86 | 0.063 |
