# Supplementary material for: Factors influencing QT interval prolongation during rifampicin-resistant tuberculosis treatment: a multicenter real-world study from China
Source: BMC Infect Dis. 2025 Dec 12;26:31. doi: 10.1186/s12879-025-11896-1 (PMC12794395; doi:10.1186/s12879-025-11896-1)
Supplement: Supplementary file 4 — Supplementary Material 4. [file 12879_2025_11896_MOESM4_ESM.docx]

**Table S3 Model performance metrics for multivariable logistic regression models.**

| **Model** | **Table 3** | **Table 4** |
| --- | --- | --- |
| AUC | 0.833 | 0.820 |
| Brier score | 0.070 | 0.071 |
| calibration intercept | 0.115 | 0.103 |
| calibration slope | 1.070 | 1.060 |

Discrimination, overall error, and calibration metrics (AUC, Brier score, intercept, slope) for Table 3 Model (regimen-focused) and Table 4 Model (comorbidity-focused).
